# Transgenic Strategies for Enhancement of Nematode Resistance in Plants

**DOI:** 10.3389/fpls.2017.00750

**Published:** 2017-05-09

**Authors:** Muhammad A. Ali, Farrukh Azeem, Amjad Abbas, Faiz A. Joyia, Hongjie Li, Abdelfattah A. Dababat

**Affiliations:** ^1^Department of Plant Pathology, University of AgricultureFaisalabad, Pakistan; ^2^Centre of Agricultural Biochemistry and Biotechnology, University of AgricultureFaisalabad, Pakistan; ^3^Department of Bioinformatics and Biotechnology, Government College UniversityFaisalabad, Pakistan; ^4^National Key Facility for Crop Gene Resources and Genetic Improvement, Institute of Crop Science, Chinese Academy of Agricultural SciencesBeijing, China; ^5^International Maize and Wheat Improvement CenterAnkara, Turkey

**Keywords:** plant parasitic nematodes, *R* genes, protease inhibitors, RNAi, plant resistance

## Abstract

Plant parasitic nematodes (PPNs) are obligate biotrophic parasites causing serious damage and reduction in crop yields. Several economically important genera parasitize various crop plants. The root-knot, root lesion, and cyst nematodes are the three most economically damaging genera of PPNs on crops within the family Heteroderidae. It is very important to devise various management strategies against PPNs in economically important crop plants. Genetic engineering has proven a promising tool for the development of biotic and abiotic stress tolerance in crop plants. Additionally, the genetic engineering leading to transgenic plants harboring nematode resistance genes has demonstrated its significance in the field of plant nematology. Here, we have discussed the use of genetic engineering for the development of nematode resistance in plants. This review article also provides a detailed account of transgenic strategies for the resistance against PPNs. The strategies include natural resistance genes, cloning of proteinase inhibitor coding genes, anti-nematodal proteins and use of RNA interference to suppress nematode effectors. Furthermore, the manipulation of expression levels of genes induced and suppressed by nematodes has also been suggested as an innovative approach for inducing nematode resistance in plants. The information in this article will provide an array of possibilities to engineer resistance against PPNs in different crop plants.

## Introduction

The word ‘nematode’ comes from the Greek word ‘nema,’ which means thread. Nematodes are thread like, long, cylindrical, sometimes microscopic worms, which can be found in a variety of environments. They belong to a huge phylum of animals called ‘Nematoda’ that comprises of plant and animal parasites, as well as numerous free-living species. They are omnipresent in nature inhabiting in all types of environments and habitats (Ali et al., 2015). However, most of the nematodes are free-living and feed on bacteria, fungi or algae. Some of them invade and parasitize both vertebrates and invertebrates including human beings, thus causing serious health damage and even human death, i.e., guinea worm (*Dracunculus medinensis*) and intestinal worm (*Ascaris lumbricoides*) (Decraemer and Hunt, 2006). On the other hand, some of these worms are serious parasites of plants and result in huge crop losses. Phyto-parasitic nematodes count for 7% of total species of the phylum Nematoda from 197 genera and 4300 species that can infect a wide range of economically important crop plants including wheat (*Triticum aestivum*), potato (*Solanum tuberosum*), tomato (*Solanum lycopersicum*), maize (*Zea mays*), soybean (*Glycine max*), and sugar beets (*Beta vulgaris*) (Decraemer and Hunt, 2006).

**FIGURE 1:**
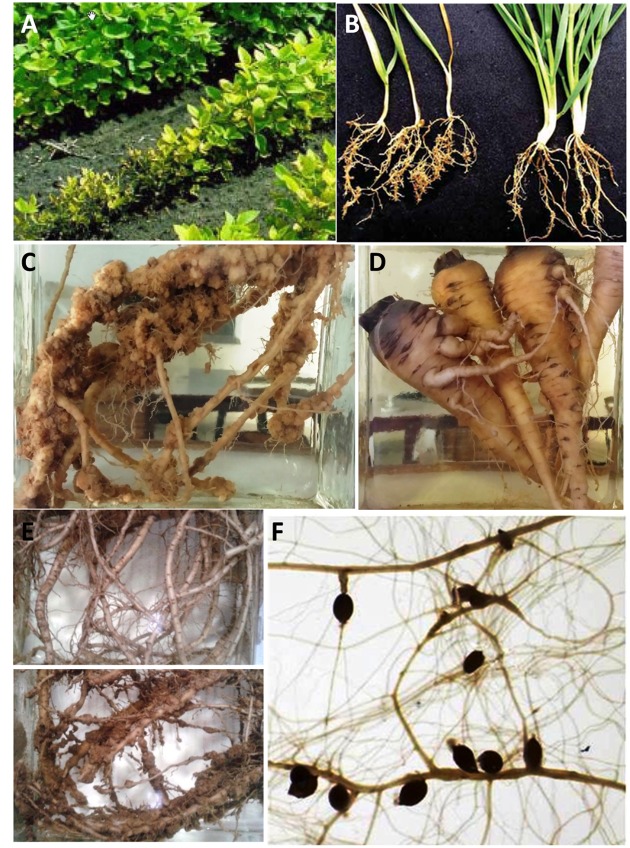
**Above-ground and below-ground symptoms from different plant species in response to nematode infections. (A)** Soybean plants infected with soybean cyst nematodes (*Heterodera glycines*) (http://www.extension.umn.edu, reproduced with the permission of Assoc. Prof. Malvick from UMN, US). **(B)** Infected and uninfected wheat plants with cereal cyst nematode *H. avenae* (Courtesy Prof. Honglian Li, China, reproduced with permission from Riley et al., 2009). **(C–E)** Roots of sponge gourd, carrots, and okra infected with root-knot nematode *Meloidogyne incognita*, respectively. **(F)** Arabidopsis roots showing development of cysts induced by beet cyst nematode *H. schachtii*.

Most of the nematodes are obligate biotrophic parasites of plants and their infection results in above-ground symptoms in plants like leaf necrosis, chlorosis, plant wilting, stunted growth, and enhanced susceptibility to other pathogens, which mostly occur in patches (Webster, 1995). Penetration of plant parasitic nematodes (PPNs) to the root systems results in the hindrance of translocation of water from roots toward areal plant parts and translocation of nutrients from leaves toward the plant roots. The developing nematodes feed on cell sap and hinder the food supply to the plant physiological systems, which results in stunted growth, yellowing and drooping of leaves. Generally, below-ground symptoms include gall and knot formation in infected roots, root lesions and necrosis and root abbreviations. **Figure 1** demonstrates the above- and below-ground symptoms on different plant species. Moreover, in addition to direct damage to crops in the form of yield reduction, these worms serve as disease transmitting vectors for viruses, thereby resulting in crucial economic and social impacts worldwide (Ali et al., 2015).

The members of family Heteroderidae (Superfamily: Tylenchoidea) are divided into three economically important genera *Meloidogyne*, *Heterodera*, and *Globodera*, which parasitize a huge number of crop plants. These are obligate endo-parasites of host plant roots, enter the plant roots as second stage juvenile larva (J2 larva) and develop specific feeding cells in the plant roots. The members of the genus *Meloidogyne*, known as root-knot nematodes (RKNs), develop giant cells (Jones and Payne, 1978). On the other hand, the cyst nematodes (CNs) that belong to the genera *Heterodera* and *Globodera* induce a very specialized feeding cell called syncytium (plural: Syncytia) (Jones, 1981). Migratory endo-parasitic nematodes are another category that is economically important. These nematodes follow destructive mode of feeding by continuously moving through the cells of root tissues and resulting in enormous tissue necrosis (Moens and Perry, 2009). The important genera from this category of nematodes are *Pratylenchus*, *Radopholus*, and *Hirschmanniella*. Moreover, *Aphelenchoides*, *Ditylenchus*, and *Anguina* are the main genera that infect above-ground plant parts like leaves, stem, and grains, respectively.

In the last two decades, our understanding of plant–nematode interactions has increased significantly. The first genome sequences of two root-knot nematodes species, *M. incognita* (Abad et al., 2008) and *M. hapla* (Opperman et al., 2008), have been described, which were significantly different from genome of the free-living nematode *Caenorhabditis elegans*. Both *M. incognita* and *M. hapla* have definite set of proteins that determine the virulence in plant species. The secretomes (set of secreted proteins through the stylets) of different PPNs have demonstrated a number of effector proteins that are involved in compatible plant–nematode interactions (Huang et al., 2003; Bellafiore et al., 2008; Caillaud et al., 2008). In response to infection of various nematodes, plant’s transcriptome resulted in increased metabolic activity in the feeding cells and suppression of defense mechanisms of the plants in most of the cases (Szakasits et al., 2009; Barcala et al., 2010; Kyndt et al., 2012; Ali et al., 2015). Most of these studies revealed considerable progress toward an understanding of plant–nematode interactions under natural conditions. On the other hand, many works have been published in the past two decades regarding the transgenic resistance in model plants, as well as the crop species using natural resistance (*R*) genes, proteinase inhibitors and RNA interference of nematode effector genes (Bakhetia et al., 2005; Fuller et al., 2008; Gheysen et al., 2010; Atkinson et al., 2012). In this article, we have focused on the most recent literature on PPNs with the emphasis on the use of different conventional and transgenic approaches to manage PPNs in different plant species of economic importance.

## Management of PPNs

The management of PPNs has been a big challenge for the agricultural scientists and farming community. The control of nematodes below the threshold level is very important for agricultural sustainability and food security. Several strategies have been tried for this purpose, which includes management through cultural, chemical, biological, and transgenic means. The cultural practices for nematode management include solarization (Katan, 1981), flooding (MacGuidwin, 1993), fallowing (Brodie and Murphy, 1975), crop rotation (Westphal, 2011; Dababat et al., 2015), cover crops (Kirkegaard and Sarwar, 1998), and a combination of some of these (Singh et al., 2009). Similarly, the utilization of natural host resistance from various crop species is a preferred approach for nematode management because it is environmentally safe and cost-saving option as compared to chemical control (Williamson and Kumar, 2006; Dababat et al., 2016). In chemical control, fumigants, organophosphates, and carbamates have been used for inhibiting nematode populations in the soil. Most of the nematicides are now being banned due to their high cost and health and environment issues (Sorribas et al., 2005). Similarly, currently available organophosphate and carbamate compounds, i.e., oxamyl, fosthiazate, and ethoprophos, are at risk of withdrawal by EU Directive 91/414/EEC due to their hazardous nature (Clayton et al., 2008).

A lot of work has been carried out and going on to find biological antagonistic microorganisms like fungi and bacteria to minimize the effect of pathogens (Kerry, 1980, 1988; Rodriguez-Kabana et al., 1987; Sayre and Walter, 1991; Stirling, 1991; Sikora, 1992; Dababat et al., 2015). Sayre (1986) listed nematode fungal parasites *Dactylella oviparasitica*, *Nematophthora gynophila*, and *Paecilomyces lilacinus* along with a bacterium *Pasteuria penetrans* as important candidates for management of nematodes. Similarly, a *Fusarium oxysporum* strain, *Fo*162, has been shown promising for controlling *M. incognita* in various studies (Dababat and Sikora, 2007a,b; Martinuz et al., 2012). However, it has been found difficult to develop a biological control agent that is effective worldwide for any plant parasitic nematode. Due to high cost and health hazards, nematicides are losing their value with the passage of time thus paving the way toward the use of nematode resistance crop varieties, biocontrol and transgenic strategies for nematode management.

## Engineering Plants for Nematode Resistance

Recent advancements in biotechnological approaches have made it possible to incorporate and express indigenous and heterologous proteins from one organism to another. This has brought about new era of crop improvements after the advent of so-called “Green Revolution” in the 1960s. Genetic engineering of different crop plants has led to improvement of both quality and quantity of the produce in addition to enhancement of tolerance against various stresses. Several disease and pest resistance genes have been introduced into crop plants through genetic engineering. The important examples in this context are *Bt* cotton (*Gossypium hirsutum*) resistant against bollworms (*Helicoverpa armigera*), herbicide resistance for weed management, salinity tolerance, and rust resistance in wheat. Additionally, genetic engineering has been exceptional for enhancement of nematode resistance in plants. Here, we discuss different ways of engineering resistance genes in crop plants to suppress the nematode infection and populations in the soil below the threshold level.

In the past, several transgenic strategies have been used for enhancement of nematode resistance in plants. The resistance genes from natural resources have been cloned from numerous plant species and could be transferred to other plant species, for instance, *Mi* gene from tomato for resistance against *M. incognita*, *Hs1^pro-1^* from sugar beet (*Beta vulgaris*) against *H. schachtii*, *Gpa*-2 from potato against *Globodera pallida* and *Hero A* from tomato against *G. rostochiensis* (reviewed by Fuller et al., 2008). The overexpression of different protease inhibitors (PIs) such as cowpea trypsin inhibitor (CpTI), PIN2, cystatins, and serine proteases has been used for producing nematode resistant plants (Lilley et al., 1999). Another main strategy was the targeted suppression of important nematode effectors in plants using RNA interference (RNAi) approach. Unlike these strategies, some recent researches have suggested that nematode resistance could be enhanced in plants by modifying the expression of particular genes in syncytia (Klink and Matthews, 2009; Ali, 2012; Ali et al., 2013a,b). An overview of various transgenic strategies aimed at nematode resistance is provided in **Table 1**.

**Table 1:** Various transgenic strategies used for nematode resistance in plants.

Molecular strategy	Name of gene	Source	Trangenic plant	Effective against	Resistance response	Reference
Natural resistance genes	*Mi-1.2*	Wild tomato (*S. peruvianum*)	Tomato (*Solanum lycopersicum*)	*M. incognita*	Triggering of HR before significant establishment of giant cells on roots	Milligan et al., 1998; Williamson, 1998
	*Hs1^pro-1^*	*wild beet* (*B. procumbens*)	Sugar beet (*B. vulgaris*)	*H. schachtii*	Abnormal syncytium development leading to starvation of nematodes beyond J2 stage	Cai et al., 1997
	*Gpa*-2	Potato	Potato (*Solanum tuberosum*)	*G. pallida*	Development of stagnated and translucent female nematodes on plant roots	van der Vossen et al., 2000
	*Hero A*	Tomato	Potato	*G. pallida G. rostochiensis*	Hypersensitive response after the initiation of syncytia which become abnormal and necrotic due to degeneration of surrounding cells	Sobczak et al., 2005
	*Gro1-4*	Potato	Potato	*G. rostochiensis* pathotype Ro1	Unknown	Paal et al., 2004
	*Rhg1*	Soybean (*Glycine max*)	Soybean	*H. glycines*	Hypersensitive response leading to abnormal syncytia and necrosis due to degeneration of cells surrounding syncytia	Kandoth et al., 2011
	*Rhg4*	Soybean	Soybean	*H. glycines*	Hypersensitive response following the initiation of syncytia, which become abnormal and necrotic due to degeneration of surrounding cells this HR breaks down with the passage of time	Liu et al., 2012; Matthews et al., 2013
	*Cre loci*	*Aegilops* spp.	Wheat (*Triticum aestivum*)	*H. avenae*	Unknown	Williamson, 1998; Safari et al., 2005

Proteinase/ protease inhibitors	*CpTISpTI-1*	Cowpea (*Vigna unguiculata*)*Sweet potato* (*Ipomoea batatas*)	Potato Sugar beet	*G. pallida M. incognitaH. schachtii*	Effect on the sexual fate of newly established *G. pallida* and reduce the fecundity of females of without inhibition of growth and development of female *H. schachtii*	Hepher and Atkinson, 1992; Cai et al., 2003
	*PIN2*	Potato	Wheat	*H. avenae*	Unknown	Vishnudasan et al., 2005
	*Oc*-*IΔD86*	Rice (*Oryza sativa*)	Potato	*G. pallida M. incognita*	Reduced reproductive success of PPNs	Urwin et al., 1995, 2003; Lilley et al., 2004
			*Arabidopsis thaliana*	*H. schachtii M. incognita R. reniformis*	-do-	Urwin et al., 1997, 2000
			Rice	*M. incognita*	-do-	Vain et al., 1998
			Cavendish dessert bananas (*Musa acuminata*)	*Radopholus similis*	-do-	Atkinson et al., 2004
			*Lily* (*Lilium longiflorum*)	*Pratylenchus penetrans*	-do-	Vieira et al., 2015
			Eggplant (*Solanum melongena*)	*M. incognita*	-do-	Papolu et al., 2016
	*CeCPI*	*Taro* (*Colocasia esculenta*)	Tomato	*M. incognita*	Interferes with nematode ability of sex determination and gall formation	Chan et al., 2010, 2015
	*CCII*	Maize (*Zea mays*)	Plantain (*Musa* spp.)	*R. similis*, *Helicotylenchus Multicinctus*, and *Meloidogyne* spp.	Anti-feedant, reduces the reproductive success of nematodes	Roderick et al., 2012; Tripathi et al., 2015

Lectins	lectin *GNA*	*Snowdrop* (*Galanthus nivalis*)	Arabidopsis, oilseed rape (*Brassica napus*), and potato	*G. pallida*, *Pratylenchus bolivianus*, *M. incognita*	Decrease in number of females and galls developed on plant roots	Burrows et al., 1998; Ripoll et al., 2003

*Bt toxins*	*Cry6A*, *Cry5B*	*B. thuringiensis*	Tomato	*M. incognita*	Significant reduction in nematode reproduction	Li et al., 2007, 2008

Anti-invasion peptides	ACHE-I-7.1	*Synthetic*	Potato	*G. pallida*	Inhibits nematode acetylcholinesterase (ACHE) leading to disorientation of invading J2s	Winter et al., 2002; Liu et al., 2005; Lilley et al., 2011
	LEV-I-7.1	*Synthetic*	Potato	*G. pallida*	Results in chemodisruption of J2s and avoids invasion	Winter et al., 2002; Liu et al., 2005
	nAChRbp	*Synthetic*	Plantain (*Musa* spp.)	*R. similis H. multicinctus Meloidogyne* spp.	Disrupts chemosensory function of invading J2s	Roderick et al., 2012; Tripathi et al., 2015

Dual resistance	*CpTI* + *Oc-IΔD86*	*Cowpea and rice*	*Arabidopsis thaliana*	*G. pallida* and *H. schachtii*	Abnormal sexual development PPNs	Urwin et al., 1998
	*Oc*-*IΔD86* + nAChRbp	*Rice* + *Synthetic*	Potato	*G. pallida*	Reducedreproductive success of PPNs coupled with disruption of chemosensory function of invading J2s	Green et al., 2012
	*CeCPI* + *PjCHI-1*	*Taro and Paecilomyces javanicus fungus*	Tomato	*M. incognita*	Reduced chitin content and retardation in embryogenesis in the nematode eggs	Chan et al., 2015
	*CCII* + nAChRbp	Maize + *Synthetic*	Plantain (*Musa* spp.)	*R. similis H. multicinctus Meloidogyne* spp.	Anti-feedant and an anti-root invasion plants with reducedreproductive success of PPNs coupled with disruption of chemosensory function of invading J2s	Tripathi et al., 2015, 2017

## Plant Natural Resistance Genes

Natural resistance genes could exist in both polygenic manner and single dominant nature. The resistance conferred by host plant single dominant resistance genes, the *R* genes from plants, interacts specifically with corresponding avirulence (*Avr*) genes in the nematode, resulting in a so-called ‘gene-for-gene’ interaction. This type of interaction initiates a cascade of defense responses in the plants. A short summary of natural nematode resistance genes is recently given by Fuller et al. (2008) to provide the basis for this kind of resistance in plants.

Several natural host resistance genes have been cloned from some plant species and could be transferred to other plant species. For instance, *Mi-1.2* from tomato against *M. incognita* (Milligan et al., 1998; Williamson, 1998), *Hs1^pro-1^* from *Beta procumbens* against beet cyst nematode *H. schachtii* (Cai et al., 1997), *Gpa*-2 from potato against potato cyst nematode (PCN, *Globodera pallida*) (van der Vossen et al., 2000) and *Hero A* from tomato against *G. pallida* and *G. rostochiensis* (Sobczak et al., 2005) and *Cre* loci from *Aegilops* spp. against cereal cyst nematodes in wheat (Williams et al., 1994; Safari et al., 2005) are some examples that could be used in future to develop cyst nematode resistance in crop plants. Transgenic expression of resistance proteins also induces the expression of PR (pathogenesis related) proteins to establish nematode resistance in plants. The potato roots expressing *Hero A* gene showed high levels of several salicylic acid (SA)-dependent PR genes in the incompatible interaction with PCN at 3 dpi (Uehara et al., 2010). They confirmed that SA inducible PR-1(P4) was a hallmark for the cultivar resistance conferred by *Hero A* against PCN and that nematode parasitism resulted in the inhibition of the SA signaling pathway in the susceptible cultivars. Similar effects were found in resistant line of hexaploid wheat carrying *Cre2* gene, which showed upregulation of ascorbate peroxidase coding gene in response to cereal cyst nematode (*H. avenae*) when compared with the expression in the susceptible lines (Simonetti et al., 2010).

Another example is *Gro1-4*, the constitutive expression of which has increased resistance in potato plants against *G. rostochiensis* pathotype Ro1 (Paal et al., 2004). *Rhg1* is another natural resistance gene identified in soybean against soybean cyst nematode (SCN), *H. glycines* (Kandoth et al., 2011). A recent study has shown that map-based cloning of a gene at the *Rhg4* locus, which is a major quantitative trait locus (QTL), contributing resistance against SCN (Liu et al., 2012). In that study, the *Rhg4* mutant was analyzed through transgenic complementation, which revealed that this gene confers resistance in soybean by encoding a serine hydroxymethyltransferase. This was further confirmed through overexpression of serine hydroxymethyltransferase in soybean roots that demonstrated 45% decrease in the number of mature cyst nematode (Matthews et al., 2013). In the same study, overexpression of nine other putative resistance genes (including short chain dehydrogenase, ascorbate peroxidase, lipase, β-1,4-endoglucanase, calmodulin, DREPP membrane protein, and three proteins with unknown function) resulted in more than 50% decrease in the number of adult females in soybean roots. Similarly, *Hero A* gene gives almost complete resistance (>95%) against root cyst nematode (*G. rostochiensis*), while it provides around 80% of resistance against *G. pallida* (Ernst et al., 2002). Transgenic expression of *Hs1^pro-1^* (resistance gene against *H. schachtii*) from *B. procumbens* into sugar beet led to nematode resistance, but unluckily was linked with the genes that were negatively correlated with beet yield (Panella and Lewellen, 2007). Moreover, the *R* genes are typically effective against one or limited range of nematode species/pathotypes. Another limitation with this strategy is the development of different nematode pathotypes, which have the effectors (*avr* genes) that would not be recognized by the *R* genes (Jung et al., 1998).

## Use of Proteinase Inhibitor Coding Genes

Proteinase inhibitors/PIs are molecules, mostly protein in nature, which inhibit the function of proteinases/proteases released by the pathogens. After the attack of herbivores and wounding, a variety of proteinase inhibitors are produced into the plants. In case of PPNs, these PIs become active against all the four classes of proteinases from nematodes, i.e., serine, cysteine, metalloproteinases, and aspartic. The PIs used for nematode resistance studies are CpTI (Hepher and Atkinson, 1992), sweet potato (*Ipomoea batatas*) serine PI (sporamin or SpTI-1) (Cai et al., 2003), PIN2 (Vishnudasan et al., 2005), rice (*Oryza sativa*) cystatin (Oc-IΔD86) (Urwin et al., 1997, 1998), and some others cystatins from maize (*Zea mays*), taro (*Colocasia esculenta*), and sunflower (*Helianthus annuus*) (Fuller et al., 2008; Chan et al., 2015).

The anti-nematode potential of plant PIs was firstly described in transgenic potato expressing the serine PI, the cowpea (*Vigna unguiculata*) trypsin inhibitor (CpTI) against PCN (*G. pallida*) (Hepher and Atkinson, 1992). Similarly, the Arabidopsis plants overexpressing cystatin Oc-IΔD86 suppress both the growth and fecundity in *H. schachtii* and *M. incognita* (Urwin et al., 1997). Oc-IΔD86 was effective against different nematode species in various plants species (Urwin et al., 1995, 2000, 2003; Vain et al., 1998; Atkinson et al., 2004; Lilley et al., 2004).

Moreover, the combinations of different PIs could be helpful to couple specificity with wide range of resistance. Transgenic expression of two proteinase inhibitors, CpTI and Oc-I1Δ86, as a translational fusion protein in Arabidopsis resulted in an additive effect against *G. pallida* and *H. schachtii* (Hepher and Atkinson, 1992; Urwin et al., 1998). Other important proteinase inhibitors are sporamin (SpTI-1) (Cai et al., 2003) and PIN2 (Vishnudasan et al., 2005), which have shown good resistant response in plants.

As compared to other proteinase inhibitors, cystatins from different plant species have met the promise of enhancement of nematode resistance in a variety of crop plants (Urwin et al., 1997, 1998; Chan et al., 2010, 2015; Green et al., 2012; Tripathi et al., 2015; Papolu et al., 2016). Heterologous expression of a taro cystatin established considerable degree of resistance in tomato against *M. incognita* infection by interference with nematode ability of sex determination and gall formation (Chan et al., 2010). Recently, a dual strategy has been used to control PCN without affecting the soil quality (Green et al., 2012). In the first approach, a peptide was precisely targeted under control of a root tip-specific promoter that disrupts chemoreception of nematodes and suppresses root invasion without a lethal effect in both containment as well as under field trial (Lilley et al., 2011). In addition to this chemoreception disruptive peptide, OcIΔD86 cystatin from rice was incorporated to control the invading larvae that are able to cross the barrier of chemodisruptive peptide. This approach establishes that a combination of these genes offers distinct bases for the transgenic plant resistance to *G. pallida* without harmful impact on the non-target nematode soil community (Green et al., 2012). This rice cystatin has also shown good control in lily (*Lilium longiflorum* cv. ‘Nellie White’) against lesion nematode (*P. penetrans*) (Vieira et al., 2015).

Recently, an anti-feedant maize cystatin and an anti-root invasion synthetic peptide were transformed into plantain (*Musa* spp., cv. Gonja manjaya), individually and in combination (Tripathi et al., 2015). The field trials of the best transgenic event containing the peptide only demonstrated 186% more yield in addition to 99% control against *R. similis*, *H. multicinctus*, and *Meloidogyne* spp. as compared to non-transgenic control. Moreover, transgenic expression of proteinase inhibitor from maize and synthetic chemodisruptive peptide resulted in enhance yield and nematode resistance in plantain. Similarly, dual overexpression a taro cysteine proteinase inhibitor (CeCPI) and a fungal chitinase (PjCHI-1) in tomato under the control of a synthetic promoter, pMSPOA, (with NOS-like and SP8a elements), had negative effects on reproduction of *M. incognita* (Chan et al., 2015). This study further revealed that dual gene transformation had more inhibition of nematodes than plants transformed with a single gene. This advocated that the use of gene pyramiding could be employed for developing and improving nematode resistance in plants (Chan et al., 2015; Tripathi et al., 2015, 2017).

Very recently, a modified rice cystatin (Oc-IΔD86) was expressed in the roots of eggplant (*S. melongena*) under the control of the root-specific promoter, TUB-1 (Papolu et al., 2016). Five putative transformants containing this cystatin exhibited detrimental effects on both the development and the reproduction of *M. incognita* in eggplant. In that study, a single copy transgenic event showed 78.3% reduction in reproductive success of *M. incognita*. This concludes that proteinase inhibitors are potential candidates for induction of nematodes resistance in a variety of crop plants to increase crop yield and minimize the damage caused by these parasitic worms.

## Nematicidal Proteins

These proteins could be characterized as anti-nematode proteins because these are directly involved in inhibiting the nematode development on the plants. Lectins, some antibodies, and *Bt* Cry proteins are some examples of these proteins. The toxicity of ***lectins*** is characterized by their ability to obstruct intestinal function of organisms exhibiting or ingesting them (Vasconcelos and Oliveira, 2004). The defense mechanism conditioned by the lectins is vital as several lectins bind with glycans (Peumans and van Damme, 1995). Overexpression of a snowdrop (*Galanthus nivalis*) lectin GNA driven by cauliflower mosaic virus promoter (CaMV35S) has been exploited to exhibit anti-nematode activity in several plants, i.e., Arabidopsis, oilseed rape (*Brassica napus*), and potato, in response to RKNs, CNs and lesion nematodes (Burrows et al., 1998; Ripoll et al., 2003).

***Plantibodies*** are the antibodies expressed in plants and also potential candidates for the development of nematode resistance. These are important because the establishment of a compatible plant–nematode interaction engages a series of processes against which plantibodies may be directed. RKNs and CNs depend on secretions of their pharyngeal glands to mimic re-differentiation of plant cells into specialized nematode feeding sites like giant cells or syncytia. Direction of plantibodies opposite to the active proteins from these secretions could be attenuated to suppress the parasitic ability of the nematode. There are only a few reports available in the literature regarding the use of plantibodies for nematode resistance (Fioretti et al., 2002; Sharon et al., 2002).

In addition to lectins and plantibodies, different variants of ***Bt toxins*** (Cry proteins) derived from *Bacillus thuringiensis* have shown promise to induce plant resistance against nematodes. These variants of *Bt* toxins are, however, more frequently used against phytophagous insects. *Bt* toxin was firstly used as an anti-nematode protein by Marroquin et al. (2000), when *C. elegans* was exposed to Cry5B and Cry6A which resulted in the reduction in nematode fertility and viability. The PPNs use feeding tube at the stylet orifice while feeding on the plants roots. The feeding tube operates as molecular sieve, allowing the uptake of certain molecules and excluding the others. The ultrastructure of feeding tubes from root-knot and cyst nematode differ which is based on the observation that root-knot nematodes are able to ingest larger proteins compared with cyst nematodes (Hussey and Mims, 1990; Böckenhoff and Grundler, 1994; Urwin et al., 1998; Sobczak et al., 1999; Li et al., 2007). Transgenic expression of 54 kDa Cry6A and Cry5B proteins in tomato hairy roots affected the reproduction of root-knot nematode *M. incognita* (Li et al., 2007, 2008). Western blotting technique showed that the 54 kDa Cry6A protein was shown to be ingested by *M. incognita*. On the other hand, this large protein could not be ingested by cyst nematodes (i.e., *H. schachtii*) due to small orifice of the feeding tube having the size limit up to approximately 23 kDa (Urwin et al., 1998). This limitation severely restricts the agronomic application of these toxins against PPNs.

## Chemodisruptive Peptides

Plant parasitic nematodes are highly dependent on their chemoreceptive neurons to sense distinct chemical stimuli for invasion into the plants. Nematodes use acetylcholinesterase (*AChE*) and/or nicotinic acetylcholine receptors for proper functioning of the nervous system. Chemodisruptive peptides are another important strategy to minimize the invasion of PPNs into the plant roots. Two peptides have been shown to bind with these receptors to inhibit their proper function (Winter et al., 2002). Both of these peptides disrupted nematode ability of chemoreception by blocking their reaction to chemical signal at very minute concentrations of up to 1 nm. Transgenic potato plants expressing a secreted peptide that inhibited nematode *AChE* leading to disorientation of invading nematode *G. pallida*, which resulted in a 52% decline in the number of female nematodes (Liu et al., 2005). The peptide is considered effective after its uptake from chemoreceptor sensillae through retrograde transport along nematode neurons to cholinergic synapses. Costa et al. (2009) has demonstrated that cyst nematode acetylcholinesterase gene (*AChE*) is expressed in chemo-and mechanosensory neurons of *C. elegans*, which further supports this hypothesis. Similarly, Wang et al. (2011) reported indirect evidence to support the mechanism by which such peptide disrupts chemosensory function in cyst nematodes. The peptide exhibits disulphide-constrained 7-mer with the amino acid sequence CTTMHPRLC that binds to nicotinic acetylcholine receptors. Incubation in the peptide solution or root-exudate from transgenic plants that secrete the peptide disrupted normal orientation of infective cyst nematodes to host root diffusate.

Moreover, chemosensory disruptive peptide that inhibits *AChE* has recently been expressed under the control of the constitutive CaMV35S promoter and the root tip-specific promoter in Arabidopsis and potato plants, where it confers resistance against *H. schachtii* and *G. pallida* (Lilley et al., 2011). This root tip-specific promoter from Arabidopsis gene (*MDK4-20*; *At5g54370*) directed expression of the nematode repellant peptide only at the sites of cyst nematode invasion and has shown strong level of resistance against PCN. This strategy has now been combined with the transgenic expression of a rice cystatin in potato to maintain high level of resistance against PCN without affecting soil quality (Green et al., 2012). By using the same technique, The International Institute of Tropical Agriculture (IITA), in partnership with the University of Leeds, UK, developed transgenic plantain for nematode resistance using maize digestive protease inhibitor cystatin and synthetic nematode repellent peptides (Roderick et al., 2012; Tripathi et al., 2013). Furthermore, pyramiding of cystatins and chemodisruptive peptide into different crop plants has shown high degree of nematode resistance and enhanced crop yields against root-knot nematodes (Chan et al., 2015; Tripathi et al., 2015, 2017).

## Utilization of RNA Interference to Suppress Nematode Effectors

**FIGURE 2:**
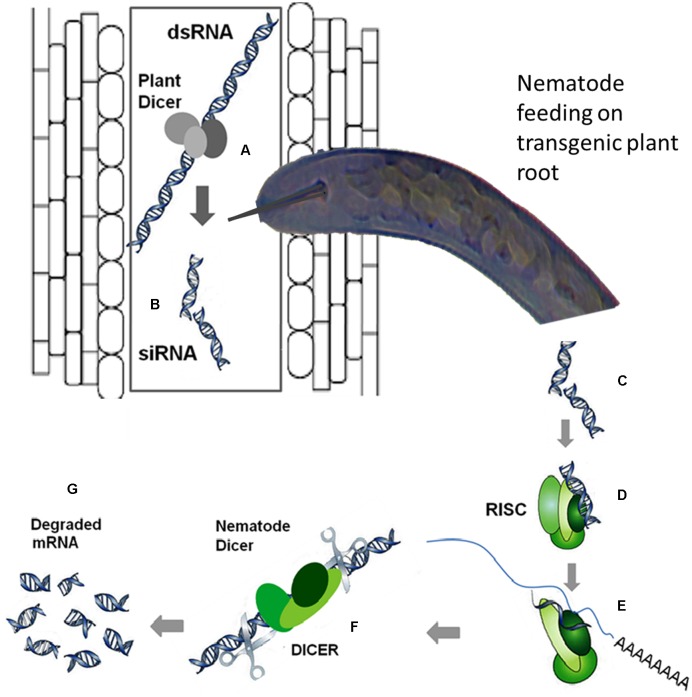
**The mechanism of RNA interference (RNAi), the double-stranded RNA (dsRNA) is processed by the plant dicer enzyme (A)** into **(B)**. Once the dsRNA is uptaken by the nematode from the plant cell while feeding **(C)**, the processing from dsRNA to short interfering RNA (siRNA) can be executed by the nematode dicer. Then, the siRNA is recognized by the RNA-induced silencing (RISC) complex of the nematode **(D)** and its unwinding into sense and antisense strands takes place. A proportion of the RISC complex loaded with the antisense strand interacts with the corresponding mRNA in the nematode **(E)** as a result the mRNA is cleaved by the RISC **(F)** and subsequently degraded **(G)**. Moreover, the targeted mRNA can be made double-stranded after binding of the siRNA, and this dsRNA is then processed to produce additional siRNAs, intensifying the initial silencing signal (Gheysen and Vanholme, 2007).

RNA interference has emerged as a very useful tool for gene-silencing aimed at functional analysis of different genes by suppressing their expression in a wide variety of organisms including PPNs. In this strategy, the nematodes uptake double-stranded RNA (dsRNA) or short interfering RNAs (siRNAs) from the plants expressing these RNAs, which elicit a systemic RNAi response in nematodes. A schematic diagram elaborating the mechanism of *in planta* RNAi is shown in **Figure 2**. The transgenic expression of dsRNA targeting a specific nematode effector gene could be handful to suppress the expression of that effector gene, which is crucial for infection process. There are many review articles mushroomed out recently emphasizing the usefulness and application of RNAi technology to induce nematode resistance in plants by silencing the expression of nematode effectors mainly (Gheysen and Vanholme, 2007; Lilley et al., 2007; Fuller et al., 2008; Rosso et al., 2009; Maule et al., 2011; Tamilarasan and Rajam, 2012). In a recent review article most of the aspects of the RNAi application in nematode resistance are reviewed (Lilley et al., 2012). Lilley et al. (2012) have reviewed various features ranging from *in vitro* assays with *C. elegans* to delivering RNAi *in planta* as an important strategy for crop protection against cyst nematodes.

Recently, Youssef et al. (2013a) have tested this approach by silencing the *H. glycines* gene *HgALD*, encoding fructose-1,6-diphosphate aldolase important in the conversion of glucose into energy and actin-based motility during parasite invasion into its host. Transgenic soybean roots expressing an RNAi construct targeted to silence *HgALD* revealed 58% reduction of females formed by *H. glycines*. Very recently, Tripathi et al. (2017) have reviewed the application of RNAi for the enhancement of nematode resistance by suppression of important effector proteins. Nevertheless, RNAi has become an established experimental tool for the enhancement of resistance against PPNs and also offers the prospect of being developed into a novel control strategy when delivered from transgenic plants.

## Other Strategies for Nematode Resistance in Plants

The manipulation of expression of genes from the plants could also be useful for induction of resistance against cyst nematodes. Transgenic plants that overexpress genes correlated with resistance or have silenced genes which are important to syncytium development and nematode success are two obvious areas to explore (Klink and Matthews, 2009; Ali, 2012). This could be achieved by using precise delivery of transgene into the feeding sites as the constitutive overexpression or suppression of a particular gene could have detrimental effects on the growth and development of the plants (Ali et al., 2013b). For this purpose, promoters that are specifically expressed in the feeding sites of PPNs, i.e., *Pdf2.1* or *MIOX5*, could be used (Siddique et al., 2009, 2011). Although constitutive promoters can deliver the gene of interest or suppress the genes which are important for compatible interaction (Ali and Abbas, 2016), however, it could be lethal for the plants to silence the genes which are also important for other physiological processes in addition to establishment of the nematodes. The promoters of the genes like *Pdf2.1* or *MIOX5* are specially and highly expressed in syncytia and several studies have shown the utility of these promoters to overexpress and silence the genes of interest specially into the feeding sites (Siddique et al., 2009, 2011; Ali et al., 2012, 2013b, 2014). In addition to syncytia specific promoters, root and root tip-specific promoters have also been used to drive site specific expression of proteinase inhibitors and nematode chemodistruptive peptides in several plant species (Lilley et al., 2011; Atkinson et al., 2012; Green et al., 2012).

The transcriptomes of various plant species infected with different nematode species demonstrated the upregulation of genes important for development of nematode feeding structures in the plant roots. The knockout mutants of two endo-1,4-β-glucanases, which were highly upregulated in syncytia, revealed less susceptibility in Arabidopsis in response to beet cyst nematode (Wieczorek et al., 2008). Likewise, an ATPase gene from Arabidopsis (*At1g64110*) was reported to be induced in syncytia caused by *H. schachtii* (Ali et al., 2013b). The knocking down of this gene using syncytia specific promoters (prom.AtPDF2.1 and prom.AtMIOX5) supported less number of nematodes.

Conversely, the nematodes are smart enough to suppress the defense mechanisms of the plants as most of the genes involved in defense related pathways were downregulated in the feeding sites revealed by plant transcriptomes in response to nematode infection (Szakasits et al., 2009; Barcala et al., 2010; Kyndt et al., 2012; Ali et al., 2015). One strongly downregulated group comprised peroxidase gene family, as out of top 100 differentially expressed genes with the strongest decrease in expression, 14 were peroxidases (Szakasits et al., 2009). Similarly, ethylene responsive transcription factor from Arabidopsis, AtRAP2.6, was one of the strongly downregulated transcripts in syncytia. This gene was driven through the constitutive promoter CaMV35S to overexpress in Arabidopsis at the global level, which resulted in reduced susceptibility in overexpression lines (Ali et al., 2013a). This also supports the debate of use of CaMV35S promoter to expression gene of interest in the syncytia induced by beet cyst nematodes in Arabidopsis roots (Ali and Abbas, 2016).

Another strategy is the expression of the genes involved in the defense pathways like camalexin and callose synthesis in plants (Hofmann et al., 2010; Millet et al., 2010; Mao et al., 2011; Birkenbihl et al., 2012). Recently, expression of *AtPAD4* under the control of FMV-sgt promoter has resulted in enhanced resistance against soybean cyst and root-knot nematodes in soybean (Youssef et al., 2013b). Expression of *AtPAD4* in soybean roots decreased the number of mature *H. glycines* females and *M. incognita* galls up to 68 and 77%, respectively. We have recently dissected the pathway of camalexin synthesis in plant–nematode interactions based on infection assays of *AtWRKY33* and *AtPAD3* mutants and overexpression lines (Ali et al., 2014). In this report, the syncytia specific overexpression of *WRKY33* resulted in the suppression of susceptibility in Arabidopsis. Similarly, the overexpression of a soybean salicylic acid methyltransferase (*GmSAMT1*) gene is found to confer resistance to SCN (Lin et al., 2013).

## Conclusion Remarks

As a consequence of enormous yield losses in crop plants imposed by the PPNs, the understanding of plant–nematode interaction is becoming of utmost importance. The nematode effectors include, for instance, cell wall degrading enzymes, the genes involved in molecular mimicry of plant genes for both compatible and incompatible plant–nematode interactions. Targeted silencing of known nematode effector proteins through *in planta* RNAi technology holds a great potential for plant resistance against various species of nematodes (Gheysen and Vanholme, 2007). A recently developed virus-induced gene silencing (VIGS) method provides a new tool to identify genes involved in soybean–nematode interactions (Kandoth et al., 2013). Similarly, the application of bioinformatics in the form of approaches like OrthoMCL could be very important for computational identification and analysis of effector proteins from PPNs (Williams et al., 1994). The development of population specific markers along with character compatibility method for diagnosis and phylogenetic inference of inter and intra specific populations of nematodes could also be done (Phiri et al., 2013). During the compatible plant–nematode interaction, nematodes are somehow able to suppress the defense related genes, the overexpression of which has led to enhanced resistance (Ali et al., 2013a). In addition to CaMV-35S promoter, several syncytia specific promoters could be used to overproduce the defense related genes in the feeding sites of the nematode to enhance resistance (Ali et al., 2013a, 2014; Ali and Abbas, 2016). This could be the interesting starting point for further studies to explicate how nematodes are able to suppress systemic plant defense mechanisms. It is concluded that the use of different transgenic strategies has shown good promise for nematode resistance. These have been helpful for reduction of nematode population on the plants on individual basis; however, by stacking all these molecular strategies together in the one plant will result in additive resistance, almost near to immunity against nematodes in crop plants.

## Author Contributions

MA conceived, designed and mainly develop the article; FA, AA, and FJ contributed toward the write up in main body of article; HL and AD reviewed and finalize the article.

## Conflict of Interest Statement

The authors declare that the research was conducted in the absence of any commercial or financial relationships that could be construed as a potential conflict of interest.
